# Medical preparedness for bioterrorism and chemical warfare: A public health integration review

**DOI:** 10.1097/MD.0000000000042289

**Published:** 2025-05-02

**Authors:** Chinyere N. Ugwu, Okechukwu Paul-Chima Ugwu, Esther Ugo Alum, Val Hyginus Udoka Eze, Mariam Basajja, Jovita Nnenna Ugwu, Fabian C. Ogenyi, Regina Idu Ejemot-Nwadiaro, Michael Ben Okon, Simeon Ikechukwu Egba, Daniel Ejim Uti

**Affiliations:** a Department of Publication and Extension, Kampala International University, Kampala, Uganda; b Health Care and Data Management Leiden University, Kampala, Uganda; c Department of Public Health, School of Allied Health Sciences, Kampala International University, Kampala, Uganda; d Directorate of Research, Innovation, Consultancy and Extension (RICE), Kampala International University, Kampala, Uganda.

**Keywords:** bioterrorism, chemical warfare, emergency response, medical infrastructure and detection technologies, public health preparedness

## Abstract

Global public health faces a major danger from chemical and biological weapon-related terrorism which requires comprehensive emergency preparedness and response strategies. This review investigates present-day public health measures against bioterrorism by focusing on an all-hazards framework which unifies traditional and nontraditional threats. The review evaluates federal programs that boost state and local health systems through funding, distribution and team-based partnerships and technological innovation. The primary emergency response elements consist of identifying outbreaks early and improving surveillance together with using state-of-the-art diagnostic tools to detect biological and chemical agents. The review emphasizes the necessity of maintaining healthcare provider education alongside preparations of full medical readiness plans as well as strategic approaches for safeguarding defenseless groups. This paper investigates resource constraints and governmental agency coordination challenges during biowarfare emergencies. The review examines nucleic-acid-based diagnostic and sensor network innovations as vital components for real-time biological agent detection systems. The review emphasizes the vital role of community involvement together with psychological resistance training in addition to continued pathogen behavior study and protection research. The review demonstrates that successful bioterrorism risk reduction depends on advanced integrated protection strategies which combine state agency collaboration with state of the art monitoring techniques and strengthened public health systems.

## 1. Introduction

**Figure 1.**
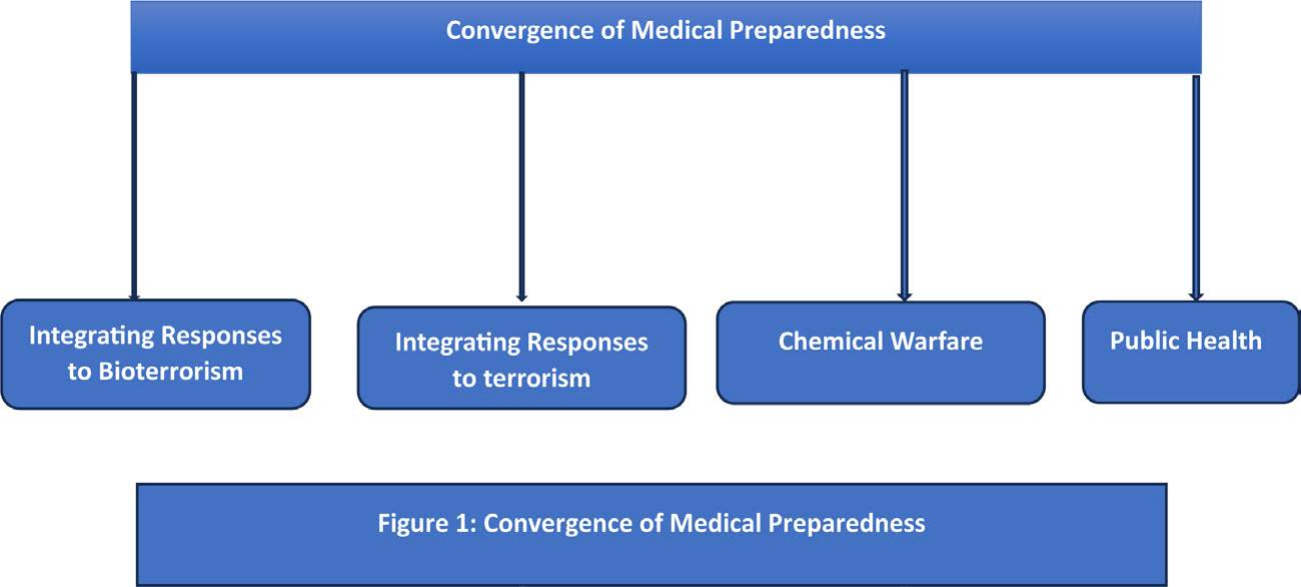
Convergence of medical preparedness.

Terrorism that involves the use of chemical and biological agents is one of the most global and steadily evolving threats to the population’s health.^[1]^ Because these acts have the potential to inflict significant casualties, traditional threats like biological agents, bacteria, and viruses, as well as chemical agents like nerve agents and ricin, should not limit emergency planning.^[2–4]^ Considering these local and public health planners in the modern world must create performance standards for emergency response plans that follow “all hazards” strategies as illustrated in Figure 1.^[4]^ This approach prevents the tendency of focusing on Weapons of Mass Destruction threats and not addressing other potential public health threats.^[5]^ New federal grant programmes have been developed to enhance the ability of state and local public health systems to prepare for and address traditional and evolving risks.^[5]^ Emergency departments are important components in this regard and, together with other elements contribute to overall preparedness and deliver acute care.^[6]^ Reporting certain diseases to the local health authorities is necessary to implement effective prevention and control measures and identify the root cause of the disease outbreak.^[7]^ Relevant examples include: outbreak of respiratory diseases is an indicator of bioterrorism hence identification of such diseases at an early stage is crucial.^[8]^ The realization that disease outbreaks may signal terrorism has therefore shifted the focus to enhance surveillance and the preparedness of public health.^[9,10]^ This has shifted attention to bioweapon identification and defense.^[11]^ In addition, the data on the use of vaccines, including preparations against anthrax, and the activity of the hotlines of the public health services will contribute to the development of state laboratories and emergency services.^[12]^ This review looks into the planning for medical disasters especially those which are bioterrorism related and other mass casualty incidents that may occur to many people.^[13]^ The importance of readiness at any level is also stressed up, including bioterrorism, chemical warfare agents and general health threats.^[14]^ Preventing the transmission of infections from first responders to other people is an important objective of public health practice.^[15]^ This is why there should be adequate pre-event vaccination, prophylactic treatment and good communication in order to increase compliance to the preventable measures.^[16–18]^ Therefore, the public health agencies should develop total preparedness and design the programmes that can assist in minimizing the effects of bioterrorism.^[19,20]^ According to experts, preparing for catastrophic bioterrorism may be useful for many potential cases, but trying to solve all possible cases at once is unrealistic.^[21]^ Instead, they advocate for a focused approach on “high consequence, low probability” events those rare but devastating bioterrorism incidents that could overwhelm healthcare systems and result in significant fatalities.^[22]^ The review revealed the following new information: The existing counter-biodefense preparedness requires enhancement. Simultaneously, threats and measures must extend beyond the scope of Weapons of Mass Destruction in a public health emergency. They also involve enhancing coordination among agencies and providing funding to healthcare workers at all levels of government to effectively respond to emergencies. Most importantly, strategies like the Smallpox Vaccination Programme and the Centers for Disease and Control (CDC’s) PulseNet System are already focusing on biological surveillance and early intervention, as recommended by the review.

## 2. Methodology

### 2.1. Research design

This narrative review employs a systematic literature review to assess medical preparedness for chemical and biological terrorism. The aim is to synthesize existing research and identify gaps in public health response strategies for chemical and biological terrorism

### 2.2. Literature search strategy

Literature Search Strategy The literature search was conducted in several databases: PubMed, Scopus, Web of Science, and Google Scholar. The search utilized a combination of keywords and MeSH terms: bioterrorism, chemical warfare, public health preparedness, emergency response, interagency cooperation, and technological innovations in identification and assessment.

### 2.3. Data collection methods

The data was obtained through a process of identified study selection and extraction of information. Important details such as study details, population, intervention strategies as well as results were documented in order to aid the analysis of the literature.

### 2.4. Inclusion criteria

Studies were included based on the following criteria:

Relevance: Concentration on bioterrorism, chemical warfare agents, and public health responder preparedness.

Time FRAME: To include only recent research findings and trends in the practise, publications from the last 20 years have been used.

Language: References to only English language material.

Type of study: Original and reviewed scientific articles, reviews, government documents, and guidelines from health organizations.

Population: Studies on public health systems, emergency response organizations and the people who are affected by disasters.

Interventions: Research on medical preparedness, actions, technologies, and collaboration among different organizations.

Outcomes: Findings on the preparedness measures and assessments, the preparedness measures’ efficiency, and the difficulties encountered.

### 2.5. Exclusion criteria

Studies were excluded based on the following criteria:

Irrelevance: Studies that do not concern bioterrorism, chemical weapons or public health.

Date of publication: All articles that were published more than 20 years ago, except for the articles that give historical background information.

Language: Non-English articles in view of translation barriers.

Non-peer-reviewed: Other publications including non-refereed articles, commentaries and letters to the editor.

Redundancy: Data or results that were duplicated or repeated in other studies that have been included earlier.

### 2.6. Strengths and limitations

Strengths: Such approach that involves the use of multiple databases provides a general view of the available literature. Emphasis on the recent literatures ensures that the current trends in public health preparedness are captured.

Limitations: Inclusion criteria may eliminate articles that were written in languages other than English. The use of only peer-reviewed articles does not consider the contributions from the professionals and other sources of information known as gray literature.

### 2.7. Ethical considerations

As this review does not involve the use of human participants, ethical clearance is not necessary. However, all the included studies were critically analyzed against set ethical standards of research.

### 2.8. Analysis techniques

Data were analyzed using the thematic analysis method in order to determine the major patterns of medical preparedness for chemical and biological terrorism. To reduce the risk of synthesizing weak and potentially inaccurate findings, the quality of the included studies was evaluated according to their sample size, detected and controlled bias, and validity.

### 2.9. Research question

What are the present indicators of medical readiness in chemical and biological terrorism, and how are these useful for the assessment of public health risks to threats?

### 2.10. Historical overview of bioterrorism

The belief that human pathogens causing diseases may be used not only for traditional state actors’ military and/or tactical special force initiatives on the battleground but also for quite criminally disguised objectives or geopolitical statements has only perpetuated the long-term global animosity against pestilential warfare.^[23–25]^ The vulnerabilities, communicability’s, defining features, and deadliness of severe pathogens in humans, as well as the falling costs and increasing technical caliber and accuracy of the delivery systems of such agents, have also combined in the context of postmodern societies to give a dark connotation to the idea of bacteriological warfare.^[26,27]^ In the 1990s, tremendous attention was paid, among other things, to the fact that the increase in destructive genetic engineering and “bionanotechnology” methods, together with internationalism and questions of professional integrity, enabled non-state professional biologists to use modern science with ease to construct effective biological weapons; such weapons are, for the most part, indistinguishable with dissimilar means to biotechnology and are fundamentally unimaginable medically.^[28,29]^ It emerges that if the latest poisoning is excluded, one hundred percent of the 13,591 cases of deliberate poisonings from chemical warfare agents in the next 71 years have involved all chemicals different from the chemical that was last introduced intentionally into a civilian population with a view to causing a biochemically mediated illness and death syndrome with possible later systemic complications from a chemical intruding into public water supply or in any other way.^[30,31]^ The change of any form of either category of agent regarding delivery systems and dissemination is nominally seen as converging from the civilization-preparedness perspective.^[32]^ The detection of a nascent outbreak rapidly enough to appropriately divert funds from the managing agency for critical community resources. The scenario that involves police, fire protection, paramedics, emergency medical technicians, ambulances, and hazardous materials handling among the first responders mainly focusses on public health training due to “background noise” after catastrophic occurrences such as Bhopal – accidental poison gas releases of the pre-antibiotic age of recent history.^[33]^ This does, however, mean that there is a need to ensure that proper communication takes place between health-relevant and other non-relevant federal organizations.^[34,35]^

### 2.11. Evolution of bioterrorism and chemical warfare

Bioterrorism, which belongs to “third generation warfare,” is a new form of “strategic surprise” affecting multinational level.^[36]^ As Charles Clapp has observed, the threat of devastation from bioterrorism or the use of chemical warfare agents is enormous.^[37,38]^ The public has become one of the leading clone dangers because awareness of the inherent toxins and germs has opened up the flood gates and placed an enormous strain on the American healthcare system.^[39,40]^ Despite this, several landmarks define the progress that the discipline has taken to get to the current level of awareness.^[41–43]^ Bioterrorism uses organisms and their derivatives that are toxins to cause disease in humans or animals as shown in Table 1. The development of warfare using bacteria, viruses, rickettsia, fungi, and protozoa affects people and has remained interesting for mankind since the Middle Ages.^[48,49]^ It has to be regretfully stated that the problems of biological, chemical, and public health have merged in this century.^[50]^ Greek writings classified poisons as “noxious vapors” and mentioned the use of poisons in the ancient world, such as the burning of vineyards in Midian, now in Saudi Arabia, by the Assyrian King Sennacherib in 700 B.C.^[45]^ At least the components of “poison soldiers,” also known as filth weapons, recurred in the eighth century A.D. and in World War 1.^[45]^ The increased utilization of exotic chemicals originated from World War 1 only and is classified under the minor eras in the history of the world, such as notes traced back to the pre-Christian period, the Chinese, the Thugs, and the Reconquista in Spain.^[44,51]^ The basic aspects of chemical warfare were rather well defined by the time of the First World War, and this contributed to the use of chlorine.^[46]^ Thus, we benefited (and at the same time suffered) from the transition to the use of phosgene, which demonstrated higher lethality.^[46,47]^

**Table 1:** Overview of the key historical events and developments related to bioterrorism and chemical warfare

Time/period	Event/development	References
Ancient World	Use of poisons and toxic fumes (e.g., “burning vineyards” of Midian by Assyrian King Sennacherib)	^[44]^
8th Century A.D.	Use of filth in warfare (e.g., “poison soldiers”)	^[45]^
World War I	Large-scale military use of exotic chemicals, development of chemical warfare techniques	^[44,46]^
World War I	Use of chlorine in chemical warfare	^[46]^
Post-World War I	Shift to the use of phosgene, found to be more lethal	^[46,47]^
Middle Ages	Development of warfare impacting humans with bacteria, viruses, rickettsia, fungi, and protozoa	^[48,49]^
Modern Century	Coalescing of biological, chemical, and public health concerns	^[50]^
Contemporary Era	Public awareness of dangers from bioterrorism and chemical warfare agents	^[37–40]^
Modern Era	Development of bioterrorism employing organisms and their byproducts to cause disease	^[43]^
Present Day	Milestones delineating the pathway to the current state of discipline in bioterrorism and chemical warfare	^[41,42]^

### 2.12. Current challenges in medical preparedness

The recent ascendancy of the terrorism threat environment led to an unmatched dividends of funds and attention to enhance general and specified medical readiness particularly biological warfare.^[52,53]^ The strategy of medical preparedness was to be achieved in terms of enhanced capacities that were directed mainly at enhancing medical countermeasures in response to new threats posed by the relevance of bioterrorism as shown in Table 2. Today, still no attack has occurred, but the overall planning and protection measures of public health have progressed.^[55]^ Studies, drills, and exercises have identified and begun to clarify persistent obstacles and dilemmas in developing effective medical responses to 3 avenues of harm: bio-terrorism with the most effective agents; terrorism using chemical warfare; and emergencies like growing disasters, epidemic and health care system shortage. Attempts at overcoming these difficulties are now made not only in the sphere of public health but encroaches on other areas associated with emergency and emergency preparedness.^[56]^ Thus, it is acknowledged that public health, emergency, and emergency preparedness lie at the federal, state, local, and tribal government’s discretion nevertheless, potential participation and collaboration, inaction, information, and capacities of numerous global federal agencies may become involved and be utilized both effectively and ineffectively within the range of responses to remind threats to homelands.^[56]^ The control of such federal benefits and hazards is more and more shifted to integrated and system-wide program stipulations.^[56]^ Thus, improved intra- and inter-agency coordination, cooperation, and collaboration are all the more relevant to developing targeted social, health, and medical outcomes within the United States and elsewhere, in the context of bioterrorism countermeasures as well as chemical warfare agents and public health emergencies, as part of use-facility readiness, and deliberative processes aimed at supporting the new doctrines’ stated goals.^[57]^

**Table 2:** Challenges and developments in medical preparedness

Area of focus	Current challenges and developments	References
Rise in Terrorism Threat	Unprecedented commitment of funds and attention to improve medical preparedness related to biological warfare	^[52]^
Medical Preparedness Goals	Increased capabilities aimed at improving medical responses to bioterrorism risks	^[53]^
Maturity of Public Health Planning	Public health planning and responses have matured despite no attack coming to fruition	^[53]^
Identified Obstacles and Dilemmas	Studies, drills, and exercises have identified persistent obstacles in medical responses to bioterrorism, chemical warfare, and public health emergencies	^[54]^
Broader Emergency Preparedness	Efforts to address difficulties now extend into broader emergency and emergency preparedness domains	^[54]^
Government Roles	Public health, emergency, and emergency preparedness are managed by federal, state, local, and tribal governments	^[55]^
Federal Agencies’ Role	Actions and capabilities of various federal agencies worldwide can impact responses to homeland threats	^[55]^
Management of Federal Responses	Integrated and system-wide program requirements are increasingly used to manage federal benefits and pitfalls	^[56]^
Coordination Efforts	Evolving intra- and inter-agency coordination, cooperation, and collaboration are crucial for guiding medical responses	^[57]^
Goals of New Doctrines	Efforts are focused on use-facility, readiness, and adherence to new doctrines in response to bioterrorism, chemical warfare agents, and public health emergencies	^[57]^

To understand interagency coordination in public health and medical preparedness, it is necessary to first discuss 2 basic forms of governmental organization: the one with a lot of free-standing and self-actuating governmental sectors apart from departments having both inspector and operator controversies, and the other, which is coherent and unambiguous in terms of command and control.^[58]^ In the former type of governmental structure, the agencies operate within the framework of anarchy. They may be obliged to exchange information and goals in the foreseeable future; they may also need to support one another to some extent.^[59]^ They may even have to seek a green light before doing something in the game, even though it is proverbial. Yet, there is no expectation that they will coordinate, not to mention collaborate, their efforts.^[60]^ Indeed, some of the most relevant ideas are presented by political scientist Paul Peterson. This is because Peterson explained the internality of the politics and ideologies of those working in public, semipublic, and fully private organizations.^[61]^ Considering the fact that the 4 mentioned agencies could sometimes exercise power – possibly life or death – over individual citizens, it was rather naïve to expect that they would have a natural resistance to the imposition of winning.^[62]^ This problem is further compounded by federalism to the extent that the travel expenses of the respondent would be reimbursed in the normal course of his activities. Several federal, state, and other local agencies will have to function under the forced circumstances of a bio-event. This is particularly the case in view of the fact that U.S. public health involves both the national and local systems.^[63]^ The greatest amount of public health, however, is practiced at the state and local levels. These are agencies that sometimes do not only have to communicate with other organizations in the realm of public health but also “water, fire, and law enforcement agencies.” In the attempt to define a meta-agency above all these institutions, it is crucial to systematically understand the type of work that arises out of complex interagency relations. For Americans, such a project used to be referred to as “revolting and transgressed.”^[64]^ In effect, the end-use of terrorist organizations that have no summer and nations of rogue regimes with weapons of mass destruction also requires adjustments in medical preparedness. Practically, however, there are serious challenges to integrating responses to the 3 main threats associated with CBW: bio-terrorism, chemical warfare and new generation diseases.^[65]^ In relation to all 3, a sustainable remedy to the current challenges that face us stems from eradicating the primary causes of each. Of all these causes, perhaps the most original one is the present status of the country’s public health system. Because of extensive changes in regulatory, administrative, and social spheres, state and local public health agencies are in a diabolical state now than in previous decades.^[66]^ In an integrated approach to medical readiness, there are 2 primary functional goals: fostering the expansion of the population-based preparedness training and enhancing the framework of public-health degree.^[67–69]^

### 2.13. Public health infrastructure

Public health faces substantial personnel and financial constraints in its efforts to address bioterrorism. The complex communications already support its public health programs. Several initiatives have emerged. These are designed to augment public health infrastructure in development.^[70]^ For instance the Smallpox Vaccination Program includes dollars and data for states to hire personnel. It also supports Information Technology work and needs such as workforce training and geographical management information systems. These are part of the National Electronic Disease Surveillance Network.^[71]^ The CDC’s PulseNet System is developing the files and data capacity to support Global Emerging Infections Surveillance laboratory data. This data comes from its collaborating agencies into PulseNet. Health Alert Network is working on technical connections with CDC’s National Health and Environmental Effects Institute. It seeks to extend electronic data collection and analysis capacity to all FHPTM’s programs. This process is directly developed to buy in part with CDC funds.^[72,73]^ The primary output of environmentally sustainable biodefense response will not be diagnostic tests and therapeutics developed to meet the threat and force of bioterrorism agents.^[74,75]^ Instead, these responses will focus on enhancing and optimizing our public health system. They will improve infrastructure emphasizing the area of greatest need.^[76]^ As stated by the Assistant Secretary for Public Health Emergency Preparedness, “Our response to bioterrorism is intended as recognition of ongoing public health efforts as coronavirus eradication of disease and Japanese encephalitis.” Any success in developing and adapting infrastructure for response will likely provide victims and treatment of millions affected with chronic connective tissue disease.^[77,78]^ The changing profile of public health into chronic disease amelioration and prevention of development rather than the subject also legitimizes the focus of present effort. It acknowledges the epistemology of bioterrorism.^[79]^ As the various researchers and commenters in this supplement have realized developing response to bioterrorism primarily requires enhancing our Public Health/intelligence infrastructure. This enables the public to expand the infrastructure necessary to identify and appropriately respond to bioterrorism and other public health events.^[80]^ A sound public practice effort is likely to include performing research on bioterrorism agents. It should also cover chemical warfare agents that might be genetically replaced with pathogenicity. The characteristics of infectious agents such as smallpox and measles should be considered. These agents have a long incubation period before becoming contagious.^[81]^

### 2.14. Current threat landscape: analyzing bioterrorism and chemical warfare agents

**Figure 2.**
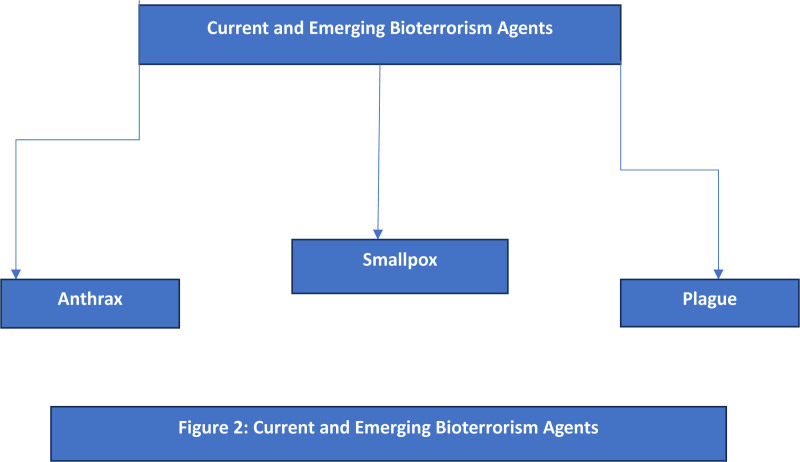
Current and emerging bioterrorism agents.

At present bioterrorism and chemical warfare are considered major threats because of the probability of catastrophic illness they may impart. They also have the ability to cause widespread panic and fear among civilians.^[82,83]^ The potential to conduct these types of warfare has increased significantly. This is due to the availability of information on the internet and the knowledge of advanced technology by medically or non-medically trained groups.^[24,84]^ Major risk factors in a scenario of biological war or bioterrorism relate to the potential of the agent to cause an outbreak of disease. They also pertain to the ability of the agent to transmit from one person to another once infection occurs.^[85,86]^ The Romans and Greeks contaminated their enemies’ water supply with decomposing animals. Infamous of all was the Chinese who smeared their enemies’ arrows with dried-up and disease-ridden corpses.^[87]^ During the crusades, bodies of those infected with skin ailments were launched to the enemies’ walls using catapults. In more recent times rifles supported by chicken feathers, which had been contaminated with smallpox were given to groups of Native American Indians by the British.^[88]^ Now in scientific age, terrorist acts with biological and chemical agents are being more widely discussed. From Islamic jihadist groups and extreme eco-terrorist organizations. To local mafia families the possibility of facing danger posed by contagious and lethal agent is becoming a concern.^[89]^ While historically only select few organisms may possess capability of being used as bioterrorism weapons the stark reality is that microbial world is vast. It contains a wide, diverse array of organisms able to cause disease and death in humans plants and animals Figure 2. In addition, advancements in genetic engineering are increasingly providing utilization of previously benign organisms for bioterrorism use.^[90,91]^ The number of organisms at present considered to be of potential bioterrorism use is relatively small. However, the capacity for mass killing and widespread societal disruption with some organisms is high.^[92]^ Bioterrorism agents therefore are biological agents that in hands of humans, represent a risk to public health.^[93]^ The use of bioterrorism agents during warfare is recognized as biowarfare. The use of bioterrorism agents by a subnational group to introduce fear and terror in a population is recognized as bioterrorism.^[94]^ Considering the extreme consequences of use of bioterrorism agents most of the efforts worldwide have been aimed at preventing the use of these organisms during warfare or terrorism.^[95]^ Extremely high containment labs have been established. Staggeringly sophisticated capabilities developed to detect the presence of these organisms respond to incidents involving bioterrorism agents and treat the exposed populations.^[96]^ Many of the technologies developed to address risks from bioterrorism agents are being (or can be) utilized for responding to naturally occurring infectious disease outbreaks. As of November 2019, naturally occurring diseases had already caused more morbidity and death than would have been caused during a militarily initiated bioterrorist attack. Interestingly although 95% to 99% of all cases of a person with chemical weapon agent are expected to be in a battlefield setting, with less than 1% of incidents associated with a terrorist event the numbers are reversed for biological agents. 95% to 99% of all incidents of human exposure expected in a non-battlefield setting.^[97]^ Anthrax is one of better-known and concurrently, most respected of biological warfare agents.^[98]^ The etiological agent *Bacillus anthracis* (*B anthracis*), a spore-forming bacterium is a Gram-positive microbial with capability of producing toxins.^[95]^ Inhalational anthrax is mostly fatal. It presents with mild, nonspecific symptoms. These would rapidly develop into deadly toxins if untreated.^[99]^ Gastrointestinal and cutaneous types are different types of anthrax. Large spreading and hemorrhaging lesions might be seen in cutaneous anthrax disease.^[100]^ Trauma may cause cutaneous form of the disease. This suggests effective agent for terrorism. According to animal aerosol experiments, social or worker contacts might be infected by individuals who are ill but do not realize it. Nearly all aerosol-disseminated *B anthracis* infections progress from insignificant joint without lymphadenopathy to massive mediastinal or hilar lymphadenopathy. Both have mediastinitis.^[101]^ The spores of deceased are concentrated in lung alveoli or peripheral tissues. Individuals who have received one or another licensed anthrax vaccine should take some time to immunize. In addition to the antibiotics vaccine is ineligible due to its slow effectiveness and subsequent vaccine procedures. These procedures necessitate a total of 6 months to successfully conclude.^[102]^ Obtaining the pharmaceuticals used in *B anthracis* therapy for treatment is simple by the military’s retail markets. It is important to understand the fluid spectrum of drugs in some cases. This is due to naturally occurring strains of non-typeable *B anthracis* isolates.^[103]^ This is important for those who may not have worked in federal laboratories. They cannot utilize Biosafety level 3 (BSL3) techniques such as those working in biodefense laboratories.^[104]^ However, it is indispensable to have rapid diagnosis and prophylactic therapy. This therapy takes the form of drugs combined to cure *B anthracis* pneumonia post-exposure following the bacterium’s air release.^[105]^ To prevent significant death and incapacitation prophylactic use of multiple or single pharmaceuticals is necessary for the duration of mobilization, deployment and mission.^[106]^ In the event of unexplained fever or fever accompanied by dry cough military personnel should see a physician. Any history of biological weapons attack will bring back individuals.^[107]^ Once correctly detected, diagnosed and untreated anthrax pandemic resulting from potential accidental discharge of pharmaceuticals will have a minimal effect.^[108]^ When infections can be treated with antimicrobials, actual deaths will decline. In time advancements in detection and response capabilities will lead to a decrease in number of casualties. This is thanks to decades of research into biological safety measures and medical interventions.^[109]^ Smallpox also known as “first and the last plague,” is contagious disease that is unique to human species. It is caused by 2 variants of Variola virus (*Variolae majoris* or *Variolae minoris*).^[110]^ The Orthopoxvirus that causes smallpox is highly infectious. It is a potential bioweapon.^[111]^ The fatality rate is 30% (depending on the strain) Survivors often bear scars all over their bodies.^[112]^ Smallpox is thus a deadly and devastating biological weapon. It is currently classified as prohibited under Biological Weapons Convention of 1972. This drew from precedent provided by international agreement to renounce use of poison gas in warfare. In 1980 World Health Organization declared smallpox eradicated. This followed the success of global vaccination campaign that began in 1967.^[113]^ In addition to having been eradicated, fact that it is very frail virus made us forget it entirely.^[114]^ Vaccinia virus is a life-attenuated virus.^[114]^ It is not causative agent of smallpox. However vaccinia was regularly employed to protect humans against smallpox from 1800 and 1980.^[115]^ The risk of smallpox outbreak is significant. This occurs in the event that insufficient people have been vaccinated and variola virus used in outbreak has significant genetic dissimilarity with vaccinia-based vaccine.^[116]^ Plague is zoonotic disease found in rodents. It is spread to humans through the bite of infected flea or by indirect human contact with infected animal tissues.^[117]^ A person with pneumonic plague can spread disease by breathing droplets containing the plague bacteria.^[118]^ Plague outbreak is more likely if the disease is widespread or endemic in local rodents.^[119]^ *Yersinia pestis* (*Y pestis*) is one of the most common and fast-acting pathogens associated with deliberate release. It produces a sparsely encapsulated form in culture that cannot be easily distinguished from other closely related enterobacterial organisms.^[120]^ There has been renewed interest in *Y pestis* as potential weapon in bioterrorism. Plague is disease that could be used as a bioweapon.^[121]^ The ease of producing and dispersion of *Y pestis* is concerning. Its capability to produce severe diseases with low infectious doses merits continued evaluation. Improved diagnostics for this organism are necessary.^[122]^ The organism itself is a particularly hardy pathogen. It is relatively stable in the external environment. This occurs under a range of climatic conditions. There are more than 60 recognized genotypes of *Y pestis*^[123]^ are recognized as having different levels of virulence. These genotypic differences have led to the concept that there may be “family” of naturally occurring “other” *Y pestis* strains. They could be more easily amenable to weaponization.^[124]^ The initial clinical presentation of plague resembles other septicemic diseases. The lethal aerosolized dose of plague under experimental conditions is typically less than 1000 Colony-Forming Units (CFU).^[125]^ The organism itself can be spread by air though secondary outbreaks occur only around primary index. Most of the human pandemics that occurred during 20th century were in tropical countries. There nevertheless was a sudden reemergence of plague in Xinjiang district in 2000.^[126]^ Most chemical agents are liquids or gases that penetrate body through the respiratory system. Sarin an extremely toxic synthetic organophosphorus compound (Table 3), was originally developed in 1938 in Germany as pesticide.^[46]^ It was methyl phosphono fluoridic acid 2-(diisopropylamino) ethyl ester. Nerve gases like Sarin, are chemical warfare agents. They have the following characteristics: they are clear colorless and tasteless liquids. These agents are sparingly soluble in water. They are used as chemical weapons. Sarin was used by Iraq in Halabja and Iran. It killed many innocent people. Most victims were women and children in the 1980s.^[130]^ Along with serious wounds many people fell ill due to exposure to nerve agents.^[44]^ Sarin has been used in warfare. It is volatile and capable of evaporating into an aerosol. This allows it to be released in airborne form. Sarin is highly toxic organophosphorus compound.^[131]^ Exposure to it results in the inhibition of enzyme acetylcholinesterase. This leads to malfunction of the nervous system. It causes symptoms that resemble those of nerve-ending stimulation.^[127]^ The symptoms include loss of consciousness. They also involve loss of control of autonomic function, asphyxiation or a combination of these. Ultimately, this leads to death as muscles fail. Sarin induces 3 major symptoms: mucous secretions bronchoconstriction and loss of nervous system function.^[128]^ Inhalation of Sarin vapor can cause these symptoms. It can induce excruciating death in a person within 10 minutes. It is classified as non-persistent nerve agent with low persistence and low toxicity properties. The lethality is influenced by the amount of exposure and the protection of skin and lungs.^[129]^ Nerve agents are among deadliest and most notorious of the class of chemical weapons. The prime reason for severity of this class of chemical toxins is they inhibit once introduced into human body, the necessary functioning of signaling enzymes.^[132]^ These enzymes are required for numerous bodily functions. This includes contraction and relaxation of body’s muscles. This lack of muscle control leads to many pronounced and unpleasant central nervous system symptoms.^[133,134]^ These symptoms include sweating salivation, bronchospasm hypertension, miosis vomiting, muscle fasciculation and low heart rate. Quick deployment of medical aid at the appearance of these symptoms is crucial. It can be lifesaving. The 2 cornerstone drugs for medical treatment of nerve agent exposure are atropine and oxime-based enzyme reactivators. Nerve agents comprise several generations of organophosphate inhibitors of acetylcholinesterase a key enzyme involved in termination of synaptic transmission. Irreversible inhibition of acetylcholinesterase occurs. This is followed by increasing levels of acetylcholine at cholinergic junctions. These changes lead to typical signs and symptoms associated with nerve agent poisoning.^[135]^ Nerve agents can be released into atmosphere or ingested by victims. In both cases, prompt and efficient recognition and identification of suspected agents would ensure better chances of achieving good outcome following exposure. This is due to ability to provide proper antidote molecules.^[136]^ The blister agents are organic compounds targeted primarily at white blood cells and potentially other rapidly dividing cell types. Blistering only occurs when the agent can penetrate the targeted cells.^[137]^ One of most toxic agents is sulfur mustard (SM). Sulfur mustard is an oily liquid that is not soluble in water.^[138]^ It has no odor in its pure state. However, impurities give it a distinctive odor. Mustard has a delayed onset of vesication.^[139]^ This delay is affected by concentration temperature, purity skin thickness and individual susceptibility. SM can cause severe damage to eyes, respiratory and skin tissue.^[140]^ The agent has an irritative effect on the eyes and respiratory system. Loss of eyesight may be result of severe injuries or negligence with decontamination. Mild skin contamination or self-decontamination is associated with rash on skin. Severe contamination can lead to debilitating skin burns and blistering.^[141]^ A similar agent belonging to another class known as lewisite, causes rapid conjunctivitis. It also induces strong irritation of the respiratory organs and blistering of the skin.^[142]^ It is treated similarly but there is no antidote. This makes treatment a very long process. During treatment, the natural decontamination process of the body may be only protection for the patient in case skin decontamination is impossible. Adequate protection for the medical and rescue staff is crucial.^[143]^ It should be implemented similarly to the mustard-treatment scenario. For large vesicated areas detachment or aspiration of the vesicae might be necessary to relieve the blister-preformed stress on the surrounding tissue. This helps promote further healing.

**Table 3:** Characteristics and effects of Sarin as a chemical warfare agent

Characteristic	Description	References
General Form	Liquids or gases that penetrate the body through the respiratory system	^[44]^
Specific Agent Example	Sarin	^[44]^
Development	Developed in 1938 in Germany as a pesticide	^[46]^
Chemical Composition	Methylphosphonofluoridic acid 2-(diisopropylamino)ethyl ester	^[46]^
Physical Properties	Clear, colorless, tasteless liquid, sparingly soluble in water	^[46]^
Historical Use	Used by Iraq in Halabja and Iran in the 1980s, causing numerous casualties	^[127]^
Effects of Exposure	Serious wounds and illness due to exposure	^[44]^
Volatility	Capable of evaporating into an aerosol and being released in airborne form	^[128]^
Toxicity	Highly toxic organophosphorus compound	^[44]^
Mechanism of Action	Inhibits the enzyme acetylcholinesterase, leading to nervous system malfunction	^[127]^
Symptoms of Exposure	Loss of consciousness, loss of control of autonomic function, asphyxiation, mucous secretions, bronchoconstriction, loss of nervous system function	^[128]^
Onset of Symptoms	Inhalation of vapor can cause symptoms and death within 10 minutes	^[129]^
Persistence Classification	Non-persistent nerve agent with low persistence and low toxicity properties	^[128]^
Lethality Factors	Influenced by the amount of exposure and protection of skin and lungs	^[129]^

### 2.15. Potential impacts on public health

**Figure 3.**
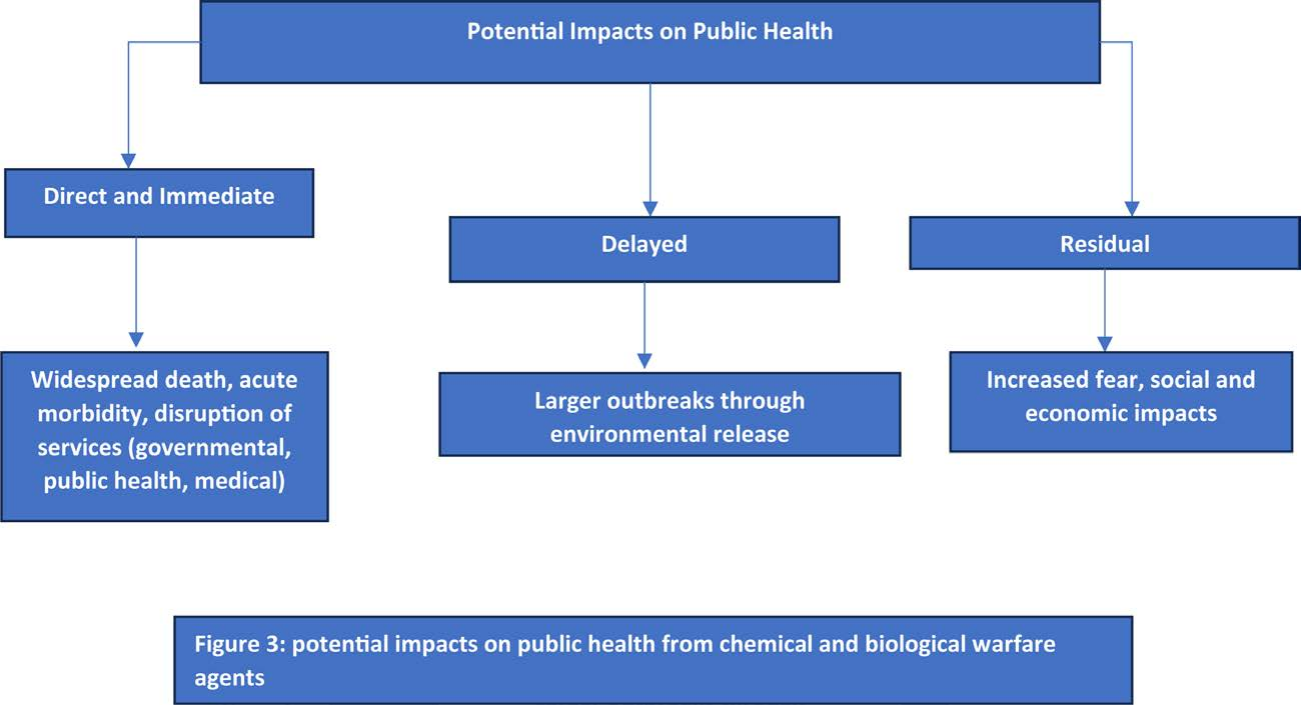
Potential impacts on public health from chemical and biological warfare agents.

The public health impacts of chemical and biological warfare agents may be direct and immediate delayed or residual as described in Figure 3. Certain agents have potential to cause widespread death acute morbidity and disruption of governmental, public health and medical services.^[143]^ Some agents may be spread from person to person. Others could cause much larger outbreaks through environmental release.^[144]^ In addition both chemical and bioterrorism agents may result in increased fear. This fear leads to social and economic impact disproportionate to actual number of environmental releases or human cases produced. Consequently these agents are known as weapons of mass disruption.^[145]^ For example the 2001 anthrax attacks resulted in 11 human cases of inhalational and cutaneous anthrax. There was mass dosing of “worried well.” There was subsequent public and social stigma associated with places of known contamination.^[146]^ This was accompanied by the closure of United States Capitol and Senate office buildings when environmental contamination was identified. It also resulted in single, indirect fatality from bacterial toxin sprayed at national media outlets. The 13-barreled drum of contaminated mail and known presence yet lack of identification of attacker or motivator created significant public fear throughout United States.^[146,147]^ Since anthrax attacks public health preparedness has increased. Global public attention on potential threat posed by both bioterrorism and chemical warfare agents has also increased. Our experience to date however is limited. It has been limited to relatively small impacts.^[144]^ These impacts are experienced from managing naturally occurring outbreaks or chemical accidents. It also includes information known from clinical and epidemiologic studies conducted with chemical and biological weapons surrogates. This includes early biotechnology products developed before enactment of international regulations or national laws governing their development and use.^[146]^ Indeed, Food and Agriculture Organization of the United Nations World Health Organization and World Organization for Animal Health (known as Tripartite) have identified high-priority pathogens and toxins that could be used as biological weapons.

### 2.16. Immediate health effects

This business’s concern that has risen each time people hear about bioterrorism and other health calamities is security of life specifically that of family members.^[10,147]^ This is a prime issue. It directly associates with the country’s public health officials and other specialists in the sphere of safety. The direct health impacts of peoples’ accidental or intentional exposure to clinical chemical warfare agents, biological agents and radiological agents are described. They point to the variety of impacts. This calls for adequate awareness of them among clinicians.^[57,147]^ Surprisingly varied are chemical agents themselves. The mechanism by which harm is caused is also diverse. Hence from cellular to whole organism mechanisms of destruction are least understood mechanisms in management of mass calamities. Particular attention is paid to measures which in complete toolkit, country’s public health system must first undertake to save lives, prevent and mitigate diseases and injuries in bioterrorism attack.^[49,148]^ Courses of action to reduce frequency of communicable diseases must be established. The occurrence of pharmaceuticals for radiological occurrences or recommendations to avoid such materials from penetrating body systems to cause health issues must also be aimed at.^[10,149]^ Communicable diseases can still decrease mass casualty populations. This occurs until evaluation of clinical exposure incidents is carried out. This can happen either through potential bioterrorism mass casualties or through people possibly affected by clinical doses from low-emitting radiation or toxic elements.^[81,150]^

### 2.17. Long-term health effects

Some identified chronic consequences e.g. cancer, may manifest in the days to years after exposure. Other late effects for example, neurological deficit may take several years or even decades before they manifest. Long-term effects stem from such biological processes. These processes include the route taken by absorbed dose.^[129,151]^ They also include interaction of tissue elements with the toxicant and sustained cellular processes. Consequently LD50 of sulfur mustard differs based on absorption problems. Such problems include the site of action, dermal absorption decontamination period and skin surface area. During absorption, factors that affect rate and extent to which drug molecules reach systemic circulation include skin thickness. The amount of blood supply in a given area matters too^[148,149,151]^ Lipophilic nature of Mustard gas or Sulfur mustard (HD) makes it become stored in adipose tissue. When exposure of HD vapor takes place it quickly dissolves in blood when inhaled and remains in lung with secondary damage. In case of HD, as with most small-molecule agents it targets eyes when disease is in vapor phase.^[134,151]^ The chronicity of this profile of increased oxidative stress may take more time to manifest severely in heavier tissues. Other possible acute effects may follow tissue injury as secondary responses to exposure. This occurs after damage from exposure has peaked. Some acute effects are inherent in tissues. When toxicity reaches a certain degree cell processes get involved^[150,151]^ As for cytoxicity, non-membrane barrier sites including the eyes, lungs and esophagus will demonstrate severe irritation signs only when concentration of tested material equals or exceeds^[151]^ Many other tissues will show signs of secondary effects like inflammation. Conditions in secondary acute lesion arise when lesion occurs. If it is due to rapidly proliferating tissue, other effects of oxidative stress will manifest^[151]^ Since HD is both an alkylating agent its effect is that tumors may present later with active growth and cell cycle nonspecific additional mechanisms.^[50,152]^

### 2.18. Integrative preparedness strategies in bioterrorism and chemical warfare response

Bioterrorism and chemical warfare are among biggest threats to world homelands and people’s health^[152]^ The scope and characteristics of conduits for bioterrorism threats differ from conventional warfare. More cases of outbreaks caused by the malicious use of dangerous agents call for measures for dealing with such malignant attacks^[153]^ Fundamental elements of public health capacity are mostly the only pathway to improvement of responses to such sorts of threats. In addition to routine and normal containment of the spread of pathogenic organisms, a radical type of preparedness planning is needed. This planning focuses on combating these agents. Particularly when they are deliberately used control of fear and panic is another major issue.^[154]^ Contacts with human disease or even mere threats of invisible microorganisms such as germs will trigger more chaos in society. This chaos depends on impacts made by the germ. Top management people and community and national leaders must be sensitive to psychological outcomes of communities. These outcomes relate to reactions to bioterrorist acts. The problems and concerns that relate to bioterrorism as well as chemical warfare crises are complex. They tend to be clearly multi-disciplinary. To address the crises responses will need to come up with a composite and integrated response to the preparedness plan. Strategic cross-cutting approaches build capacities for prevention early identification instantaneous diagnosis and reporting. Sharing of information and practising an efficient response to these occurrences will assist guide United States (U.S) on the process of attaining structure for early intervention in such calamities. Counter-terrorism, critical infrastructure protection paradox readiness, positive button readiness identifier, strength personnel.^[155,156]^

### 2.19. Technological advances in early detection and monitoring of bioterrorism and chemical threats in community settings

The threat due to exposure through aerosolized or gaseous agents is distinct from threat through biological agents with intervening periods of dispersion as shown in Table 4. Bioterrorism uses biological agents that affect health of the community’s population as cases of the released pathogen. This occurs without direct transmission of infection through human contact at the episode site.^[10,15,156]^ However, chemical agents are listed as chemical weapon agents of mass destruction commercial hazardous gases or volatile organic compound in which release will result in acute toxicological impacts. People get affected or die at scene of incident.^[12,46]^ As a rule, there are essential distinctions that can be observed between biological and chemical agents. In cases of bioterrorism it is possible to wait for many hours to days before facing consequences at an acceptable rate among perpetrator as well as third party. To be considered as most likely chemical agents, however chemical warfare agents require rather fast release into given zone inhabited by people. This is necessary to provide intended impact.^[10,157]^

**Table 4:** Technological advances in early detection and monitoring of bioterrorism and chemical threats in community settings

Aspect	Bioterrorism	Chemical threats	References
Type of Agents	Biological agents causing diseases	Chemical weapon agents of mass destruction, commercial hazardous gases, or volatile organic compounds	^[10,12]^
Method of Attack	Intentional release of biological agents	Aerosolized or gaseous chemical agents	^[10,12]^
Transmission	Can have person-to-person transmission at the site of release	Causes acute toxicological effects at the site of attack	^[15]^
Detection and Monitoring Differences	Detection and monitoring significantly differ from chemical threats	Requires rapid release for desired effects	^[10]^
Risk to Perpetrator and Bystander	Many hours to days may elapse with modest risk	Immediate acute effects causing illness and death	^[10,12]^
Desired Effects	Causes disease in the community population	Causes acute toxicological effects at the site of attack	^[12]^
The time frame for Effects	Hours to days for the effects to become apparent	Immediate effects upon release	^[10]^

### 2.20. Types of chemical threats

**Figure 4.**
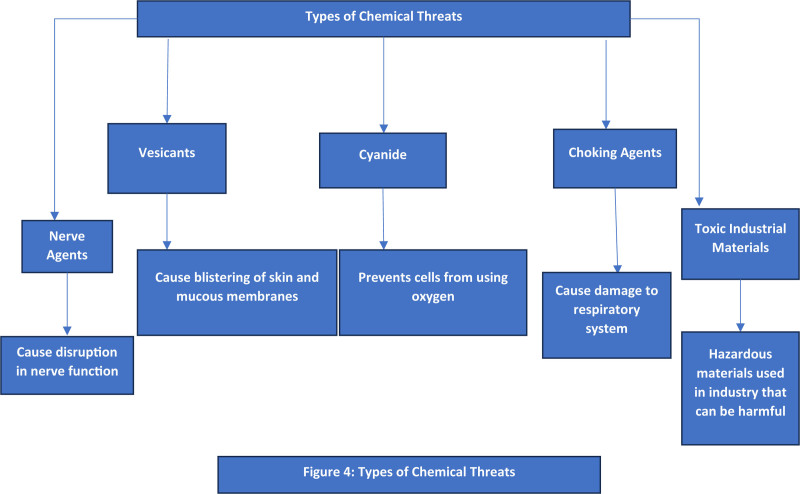
Types of chemical threats.

Although chemical agents and toxins are grouped as one category in emergency preparedness they pose distinct clinical and public health challenges as shown in Figure 4.^[157]^ The 5 primary categories of chemical threat agents are nerve agents, vesicants, cyanide, choking agents, and toxic industrial materials.^[158,159]^ Concerning bioterrorism preparedness significant limitation of traditional surveillance systems is the time lag between onset of illness and report to public health authorities.^[160]^ It is essential to enhance detection capacity at clinical level before or concurrent with notification of event to public health authorities. Such development includes widespread implementation of electronic health records. It also involves clinical decision support systems and linkage to health information systems like laboratories, electronic hospital resources and poison control centers.^[161]^ Data generated in these clinical systems can identify potential bioterrorism or chemical exposures early in fact. This can happen even before intentional release is known.^[160]^ Real-time bioterrorism and chemical threat detection systems that can continuously monitor environmental sensors. An example is the United States Environmental Protection Agency Air Quality System AQS. These systems must also be capitalized.^[161]^ Harnessing data on increased bioterrorism awareness can change scale and resolution of existing models. This focuses investments on most at-risk populations and areas of highest significance. Such a novel approach would improve prioritization of resource utilization regardless of level of preparedness assistance desired.^[88,161]^ Ultimately these infrastructure changes are practical, justifiable and highly beneficial to community health. Bioterrorism and general chemical threat preparedness are largely untreatable and only infrequently encountered.^[57,162]^ Moreover investments in general public health and preparedness measures tend to build resilience against both public health challenges and security threats. This achieves dual objectives of efficiency and improved national security.^[163]^

### 2.21. Importance of early detection and monitoring

Early detection and monitoring of serious chemical threats and bioterrorism activity has become a critical objective of the U.S. government and its defense organizations. Until very recently, national efforts were primarily focused on protecting populations, prevention, and providing a warning and response to the scale of a catastrophe, once a biological weapon was delivered or a chemical attack initiated.^[164]^ Since the 2001 anthrax mailings, the field has been energized by related evidence of prior biological and chemical attacks or operations involving others in other countries as well as by principles and computer simulations for biological warfare defense. Imported cases of A (H5N1) avian influenza, which dampened international stock markets and severely affected the business operations related to birds and bird products of countries receiving the poultry, have added to real-world concerns about the current risks of infectious disease outbreaks containing potential pathogens or a chemical attack.^[78,165]^ Certainly, the U.S. response to the intentional release of *B anthracis* spores has molded new technologies. Advances in the early detection and monitoring of *B anthracis* spores and anthrax infections are the subject of other papers in this issue.^[34,166]^ Here, we describe new and emerging technologies that can be applied in oft-used public places. These technologies make detections from built-in sensors in minutes to a few critical hours using hardware currently available or under development in the commercial sector.^[61,166]^ Immediately available technologies that could be applied to provide early detection, monitoring, or decision support for the chosen agents detected agents such as small airborne particles of a broad range of sizes and chemistries.^[163,167]^

### 2.22. Potential impact of bioterrorism and chemical threats in community settings

Terrorism poses continued threat to the security of persons in global community.^[164,167]^ The utilization of chemical agents in Syria and discovery of some chemical chemicals in several Al-Qaeda safehouses gives each community good reason to prepare for worst case scenario. Tremendous progress has been made in the areas concerning detection and response to threats.^[168]^ The ability to convey these threats to first responders in community in an as real time manner is crucial.^[168]^ Additionally diagnosing and treating those potentially exposed to them or those who have already been infected is important. In addition, while verification of such devices for both identification as well as screening for efficiency within disaster scenarios mimics field testing it makes this complex but essential bi-faceted problem.^[168]^ Bioterrorism refer to act of using living organisms to threaten well being of communities. The effects if carried out could be immense. For instance Amerithrax mailings of year 2001 impacted over 30 addresses. They led to quick shut down of US postal services.^[167]^ Postal Service processing facilities. Indeed, letters bore message: You cannot stop us. We have this anthrax.^[168]^ “You die now Are you afraid of Death to America. Death to Israel. Allah is great.” Biological exposure in 5 US states infected 22 people and caused fatal inhalation anthrax in 5 people with minimum of 10,000 people counseled to take ciprofloxacin or doxycycline as prophylaxis.^[169]^ The total cost of the attack received a high estimation.^[170]^ It is believed that more than $13 billion were necessary to counterbalance for medical, postal and environmental consequences of anthrax spore spreading attempts to recover postal facilities took years.^[171]^ The path of anthrax transport entities would require further efforts.

### 2.23. Benefits of rapid detection technologies

Blood and other biosamples often require collection and extensive preparation for analysis before chemical or biological threats can be detected or excluded. Rapid detection technologies overcome these often laborious approaches.^[172]^ They provide a more thorough analysis. This reduces both the time to detect the type of chemical or biological threat and the level. This indicates a more definitive and specific diagnosis in subjects suspected of exposure.^[173]^ It will in turn, contribute to better medical monitoring and management of exposed individuals. Additionally extended exposure monitoring and redundant diagnoses can be provided in parallel with reduced time delays – a critical advantage in time-critical care practice for exposed individuals. The recent advances in technological diagnostics and monitoring tools have enabled minimization of limitations faced in both resource availability and time-related critical applications.^[173]^ Since response time to chemical agents is crucial it is an essential element within the assessment and management of exposed individuals. A real-time detection method has to be applied to blood-circulating diseases in connection with exposure event.^[174]^ Toward this direction, we discuss within present review article the development of rapid field tests and portable instruments for detection of cancer from blood plasma and bacteria plus specific blood markers.^[175]^ Current progress in the development of novel methodological approaches is also presented. This approaches aim for direct detection of binding footprints of circulating chemical or biological agents in blood. The observed detection of binding footprints can be related to presence and association of pending health status or disease. The discussion also involves number of typical advantages and limitations of developed methods.^[175]^

### 2.24. Cutting-edge technologies for early detection

While the capabilities of transit commercial and postal peer screening are growing rapidly, these systems are only likely to be successful in identifying agents in letters small parcels, or very large releases.^[174]^ Counting on detection at a facility using these technologies means that first responders will almost certainly have to cope with initial casualties. This includes the collateral consequences of dealing with panic and people trying to leave the area. Reducing the consequences associated with bioterrorist or chemical terror releases depends on detecting such events in community well before the release.^[175]^ This is quite distinct from overwhelming pressures facing large urban post offices or transit hubs. Advances that allow for detection in ambient metro settings would be leveraged in other community locations

Three technologies – nucleic-acid-based diagnostics and environmental monitoring array-based pattern recognition and standing wave sensor architectures – have been researched as bioterrorism leads to early detection in community settings.^[176]^ Nucleic-acid-based diagnostics, such as PCR are highly sensitive and specific. They have been used successfully in Environmental Rapidly Deployable Instant Microorganism Detection and Classification system and BioloGENE systems.^[176]^ Array-based pattern recognition technologies use pattern recognition methods. These analyze the response of several relatively nonspecific sensors. Each sensor individually responds to typical combinations of volatile organic or inorganic materials.^[177]^ There are 2 or more well-thought-out methods for pattern recognition: prototype or model-based approaches and non-parametric or data-driven, more holistic approaches. Instead of depending on specific league that would inevitably be circumvented by a bioterrorist seeking merger a large number of airs. Ideally, water or wastewater sensors would allow one to use more statistical approaches to making a decision.^[178]^ Furthermore as operating environment for these sensors is not likely to be laboratory or factory, robust technologies are necessary to achieve real-world implementation. Large responses like sample pre-air conditioning are impractical. Calibration units are generally all for sensor stability. This allows unpredicted issues to arise.^[177,178]^ Array-based pattern recognition technologies currently come in 3 different types: geometric spectroscopy rapid chemical vapor detection and mass spectroscopy. Finally, standing wave sensor architectures utilize wave reflection capability of a stratified waveguide. Critically this platform technology allows for remote or unattended sensor capabilities.^[179]^ The classes each have modern cellular paradoxes. They show promise on another detection timeline instead of mechanical continuum between screening facilities and first metro response capabilities.^[180]^

### 2.25. Rapid diagnostics

One key concept that requires support and future research is the technology to engineer and manufacture highly sensitive, specific, biodefense-relevant biosensor-based rapid diagnostic tests that are suitable for use by first responders at the site of an event.^[181]^ For biodefense rapid diagnostics to become fully effective for these missions, a combined national mission to address clinical needs, to identify and resolve practical issues of needing isolated diagnostics that also must identify risk to unprotected sentinel populations, questioning how rapid the answer must be as it relates to decision making, while defining how sensitive and risk-assessing different solutions related to selection of operational false positives.^[181]^ Among the advanced concepts that are crucial to event site detection and response that use progressively sensitive and highly portable diagnostic platforms are fully integrating sample preparation techniques compatible with Laboratory BSL-2-level test performance.^[182]^ Other advanced concepts include getting a recognizable readout from a diagnostic test in less than thirty minutes, excellent sample collection techniques, integrating chemical and biological detections, and sensor fusion-based molecular and physicochemical tests, and using for most of the rapid and sentinel diagnostics.^[183]^ All of these tests must be performed at the site of an event where the technical work is hindered by wanting rapid and more specific results, while all other parameters in the diagnostic development process are sacrificed.^[184]^ While preliminary benchmark experimental studies will often define the ultimate reach of our technical capabilities, when life is at risk it forces consideration of when we are better off to settle for less stringent criteria, and associated compromises when the answer should be obtained; it is an acceptable balance between the practical clinical realities but inevitable timeliness.^[185]^

### 2.26. Sensor networks

Sensor networks are deployed to observe a given field of interest and are composed of multiple sensor nodes, wireless communication modules, and a data station or processing center.^[183]^ Energy and resource constraints often have a major role in designing sensor network systems. Each sensor node is typically composed of 4 main parts: a sensing unit, which is composed of one or more sensors; a communication unit; a power source; and a small Central Processing Unit that takes care of low-level control and communication issues.^[182]^ In addition, sensor networks offer monitoring from a large number of mobile and static configuration nodes.^[183]^ A large-scale sensor network also has the benefit of close correlation between the location of optimized software-defined radio response detectors, meaning that it is possible to accurately determine the geographic origin of a radiation source.^[184]^ This is particularly important in converged threat detection networks, where sensor networks designed to detect chemical, biological, radiological, nuclear, and explosive (CBRNE) threats work in concert. Finally, the passive and single pulse nature of the sensors leads to a low probability of detection, a major consideration in sensor deployment.^[185]^ One of the highest impact areas for the applications of sensors in the detection of CBRNE materials is security in urban environments. Sensor networks deployed in these environments aim to detect the presence of unauthorized people and ambient conditions related to criminal activities.^[186]^ They might operate unattended or in cooperation with nearby security staff, coordinating the sensed information to take proper countermeasures.^[187]^ The extended integration of sensor networks appears to be essential when, incidentally, the mere deployment of more sophisticated sensor platforms is growing rapidly.^[188]^ The next generation of military operations in urban environments Military Operations in Urban Terrain (MOUT) is expected to require an extended integration of robotics, sensors, and sensor systems. These platforms must be adequate to rapidly react to the challenges of the “emergency action” actions.^[189]^ The deployed solutions must be modular to allow easy integration of other sensor types as they become available and of additional telecommunications subsystems. For those platforms that require coverage on very large areas, we also need the ability to realize ad hoc networks of heterogeneous platforms, in which both fixed and mobile nodes cooperate to reach the objective as fast as possible.^[190]^

### 2.27. Challenges and limitations

New technological advances, the development of novel biochemo-sensors-, point-of-care diagnostics, and lab-on-a-chip and cell-phone platforms have enabled early-stage diagnostics of bioterrorism and chemical threats in laboratory settings.^[190]^ To translate these technological research advances into clinical practice in community settings, researchers face several challenges that limit current technologies used for the detection and monitoring of threats.^[191]^ These challenges and limitations include the high speed, low cost, high resolution, high selectivity, sensitivity range, field performance and stability, response latency, need for sample processing, environmental interferences and operation methods of sensors, toxic effects of samples to other cellular systems, uniformity and purity of nanoparticles, quantum yield and stability of nanoparticles, standardization for good laboratory practice, and sharing the same standards for nanoparticle characterization and measurement.^[192]^ Furthermore, challenges to early detection and prevention include public health advocacy and behavioral factors, ethical and social concerns, legal constraints including data sharing, private versus public needs, current regulations, other relevant metrics for evaluation of technology effectiveness, deployment, and global concerns about militarized use rather than public health^[193]^ Unbiased deep phenotype profiling that increases data-driven research based on continuum data from various omics, disease-signaling pathways, networks, identifiable and transcriptional signatures, other submolecular traits, or the microbiome could enable rapid diagnostics of unknown threats.^[194]^

### 2.28. Integration with existing systems

The design of complex systems typically involves not only developing working components but also integrating these systems with existing or upcoming technologies.^[195]^ Previous work can be leveraged and utilized to ensure that new systems align closely with existing systems and advance new capabilities more effectively.^[195]^ Here, we have used a probabilistic Harvard model to intercept such dual-use Fitness/Host Infection (FHI) systems originally developed to model the progression of infectious diseases and provide a lens through which surveillance system developers and policymakers can build from the current state of early biothreat detection via epidemiological surveillance to the potential future state of integrated biothreat detection via existing civilian health monitoring systems.^[196]^ Our approach is to start with a civilian health monitoring perspective as also done in the modeling of biocomplexity and the Civilian Health and Medical defense domain (CHMD) use-case descriptions defined for BioPortal3.^[196]^ The surveillance model starts with the existing geographic distribution of available health data for the Emergency Detection (ED), Veterans Affairs (VA), and North American Aerospace Defense (NORAD) systems.^[197]^ The Harvard Health Decision Science Probabilistic Model provides an abstraction of 3 existing health systems relevant to testing and lab confirmation of different biothreats within a community setting.^[78,197]^ The ED and VA systems are similar in that available medical data feeds into the local county health department, which then provides summary reports to the CDC.^[90,198]^ In 2004, the ED and VA systems accounted for 2353 of the 4500 + sensors collecting data on about 45% of the US population.^[198]^ The NORAD system, according to Simwars in December 2001, added the capability for tape-based ambulance report data to be used to get input from both federal and state EMS systems for “wiping and syndromic surveillance only.” This work is part of efforts to defend against possible thermo-mechanical bioterrorism attacks exploiting known vulnerabilities in the US terrorist infrastructure.^[199]^

### 2.29. Privacy and ethical concerns

Privacy and ethical concerns are at the heart of the issue with proprietary tools and programs, law enforcement activities, and therapeutics being suitable only on a limited basis, given a potential violation of constitutional rights. BioWatch’s impact on public health, in this respect, has the potential to be a prominent issue.^[195]^ Furthermore, the use of technologies, for example, in bacteria warning, may be seen as a breach of tribal and religious codes and so be extremely sensitive to matters of biosecurity.^[189,196]^ In principle, the use of any novel technology or existing technology in a novel application or form requires careful consideration of the ethical dimensions in the first step and the development of mitigating strategies for the possibly raised concerns in the second step.^[197]^ Factors that were previously perceived as natural barriers to the wider availability and use of highly toxic biochemical weapons, such as economic and technical complexity, difficulties, and time-consuming steps in development, storage, transportation, deployment, and the risks of detonation, are rapidly losing effectiveness.^[198]^ The potential for catastrophic effects of bioterrorism is now reflected in the development of a new scientific discipline applying biotechnology in a counterterrorism role - biosecurity. It follows that the likelihood of accidentally or deliberately releasing biological agents in the civilian population is non-zero.^[189–191,199]^

## 3. Highlights

This review highlights several critical findings regarding medical preparedness for chemical and biological terrorism:

Increased Threat Awareness and Funding: This has been in a bid to strengthen the public health systems due to the high risks posed by bioterrorism and chemical warfare. Federal guidelines encourage local and public health planners to develop performance standards that encompass “all hazards,” beyond just weapons of mass destruction.

Interagency Coordination and Response: This paper finds that interagency coordination is crucial for a strong public health response. This review is critical to the point that enhancing public health and emergency preparedness at the federal, state and local level can enhance the handling of bioterrorism. Some of the current efforts are the CDC’s PulseNet System, which has been developed in order to enable early identification and control.

Technological Advances in Detection: New tools, such as nucleic-acid-based diagnostics, and portable sensor systems, have improved the ability to detect chemical and biological agents. These advancements in technology assist in the early identification of a disease and also the necessary medical management; factors that are very vital in reducing the number of death in the event of a bioterrorism attack.

Public Health Infrastructure Challenges: However, the public health agencies still have many organizational limitations especially in terms of personnel and finances to help them sufficiently plan for bioterrorism threats. The implementation of lesson learnt from previous incidents and research on high-risk agents is still critical in enhancing these systems.

Understanding and mitigating risks: The review recognizes major biological agents, including anthrax and plague, as important threats that necessitate improved surveillance and precautions. Study of the transmission patterns of these agents is therefore important in order to devise prevention and control measures.

Integrated Preparedness Strategies: Therefore, preparedness for bioterrorism, through the integration of technology as well as community participation is important in the fight against bioterrorism. It also makes a point on management of public fear and good communication strategies as part of the overall response plan.

### 3.1. Future directions and recommendations

**Figure 5.**
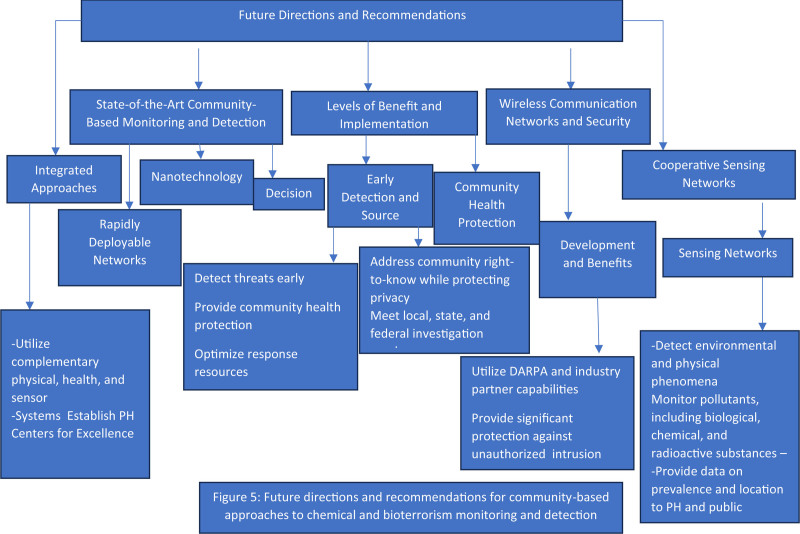
Future directions and recommendations for community-based approaches to chemical and bioterrorism monitoring and detection.

Strong, integrated approaches, utilizing complementary physical, health, and sensor systems combined with Public Health (PH) Centers for Excellence, will be required to develop state-of-the-art community-based approaches for chemical and bioterrorism monitoring and detection to protect and react to these threats Figure 5. (PH Centers for Excellence have been charged by Homeland Security Presidential Directive 10 (HSPD-10) and the National Response Plan with specific goals of enhanced chemical, bioterrorism, radiological, and nuclear weapons defense, and will provide consistency and established public health goals and expertise).^[192,195]^ In addition to rapidly deployable commercial monitoring and detection networks, in consideration and development are nanotechnology enabling multifunctional systems capable of both early biological threat detection and chemical threat detection or protection, and scalable levels of sensitivity to range from homeland defense to community needs.^[193,196]^ Such capabilities can be integrated with existing information networks and can both add and derive information to optimize response resources through the use of sound decision support systems and enable robust and efficient response optimization.^[194,197]^ The benefits of a comprehensive monitoring approach should be at 2 levels: early detection and source interrogation, and community health protection and response resource optimization.^[195,198]^ Implementation of such strategies must address both the importance of a community right-to-know to related health risks while meeting the need to protect privacy and maintain the integrity of ongoing local, state, and federal investigations. With the increasing development of wireless communication networks and the capabilities of the Defense Advanced Research Project Agency (DARPA) and their industry partners, the realized benefits of this work are expected to be obtained in the near term.^[197]^ Implementation of the discussed strategies must address all security requirements and provide significant protection against unauthorized intrusion through both passive and active measures. Such adaptive measures have to carefully adapt to changing environmental and surveillance needs.^[198]^ A rationale to address inherent issues of ownership recognition and additional information security is developed. Cooperative sensing networks can detect environmental and physical phenomena that could create threats to human and community health.^[199]^ That information can be distilled through monitoring to provide Public Health and the public with data on the prevalence and location of pollutants, including biological, chemical, and radioactivity in the community’s physical environment.^[199]^

## 4. Conclusion

Both bioterrorism and chemical warfare are issues that need proper attention since they pose a serious threat to the life of the people. The findings of this review support the need for the formulation of the medical preparedness tactics to accommodate coordination of large-scale disasters. Real-life examples of bioterrorism and chemical warfare show how these threats have developed over time and why effective health infrastructure is still very important today. Some of the issues being faced currently are lack of efficiency and coordination of federal, state and local bodies and no ideal inter agency cooperation. Some of the main strategies that have been proposed to tackle these difficulties include strengthening the capacities of public health, offering extensive training, and introducing advanced detection techniques. At the same time, the review reveals main ethical and privacy concerns arising from the use of sophisticated technologies for detection of threats and the need to find the right balance between security enhancing measures and citizens’ rights protection. Meeting medical preparedness requires an integrated approach in terms of bioterrorism, chemical warfare, and other public health threats because such events are rare, but their consequences may be severe. In other words, population-wise, through training, strengthening public health systems, and more linking can help diversify threats to its population. The review calls for constant assessment of readiness and enhancement of the preparedness strategies so that the public health systems are able to respond to the dynamic threats.

## Acknowledgments

We are grateful to Kampala International University Uganda for its support.

## Author contributions

**Conceptualization:** Chinyere N. Ugwu, Okechukwu Paul-Chima Ugwu, Val Hyginus Udoka Eze.

**Investigation:** Regina Idu Ejemot-Nwadiaro.

**Methodology:** Okechukwu Paul-Chima Ugwu, Esther Ugo Alum, Val Hyginus Udoka Eze, Mariam Basajja, Jovita Nnenna Ugwu, Fabian C. Ogenyi, Michael Ben Okon.

**Resources:** Michael Ben Okon.

**Supervision:** Chinyere N. Ugwu, Okechukwu Paul-Chima Ugwu, Esther Ugo Alum, Val Hyginus Udoka Eze, Mariam Basajja, Jovita Nnenna Ugwu, Fabian C. Ogenyi, Regina Idu Ejemot-Nwadiaro, Simeon Ikechukwu Egba, Daniel Ejim Uti.

**Validation:** Chinyere N. Ugwu, Okechukwu Paul-Chima Ugwu, Esther Ugo Alum, Val Hyginus Udoka Eze, Jovita Nnenna Ugwu, Fabian C. Ogenyi, Simeon Ikechukwu Egba.

**Visualization:** Chinyere N. Ugwu, Okechukwu Paul-Chima Ugwu, Jovita Nnenna Ugwu, Fabian C. Ogenyi, Simeon Ikechukwu Egba, Daniel Ejim Uti.

**Writing – original draft:** Chinyere N. Ugwu, Okechukwu Paul-Chima Ugwu, Esther Ugo Alum, Val Hyginus Udoka Eze, Mariam Basajja, Fabian C. Ogenyi, Regina Idu Ejemot-Nwadiaro, Michael Ben Okon, Simeon Ikechukwu Egba, Daniel Ejim Uti.

**Writing – review & editing:** Chinyere N. Ugwu, Okechukwu Paul-Chima Ugwu, Esther Ugo Alum, Mariam Basajja, Jovita Nnenna Ugwu, Regina Idu Ejemot-Nwadiaro, Michael Ben Okon, Simeon Ikechukwu Egba, Daniel Ejim Uti.
